# Balanced activation of Nrf-2/ARE mediates the protective effect of sulforaphane on keratoconus in the cell mechanical microenvironment

**DOI:** 10.1038/s41598-024-57596-9

**Published:** 2024-03-23

**Authors:** Ruixing Liu, Ruojun Ma, Xiaoming Yan

**Affiliations:** 1 Department of Ophthalmology, Peking University First Hospital, Beijing, 100034 People’s Republic of China; 2 People’s Hospital of Zhengzhou University, Henan Provincial People’s Hospital, Henan Eye Hospital, Zhengzhou, 450003 People’s Republic of China

**Keywords:** Cell biology, Diseases, Medical research, Molecular medicine

## Abstract

Keratoconus (KC) is a progressive degenerative disease that usually occurs bilaterally and is characterized by corneal thinning and apical protrusion of the cornea. Oxidative stress is an indicator of the accumulation of reactive oxygen species (ROS), and KC keratocytes exhibit increased ROS production compared with that of normal keratocytes. Therefore, oxidative stress in KC keratocytes may play a major role in the development and progression of KC. Here, we investigated the protective effect of sulforaphane (SF) antioxidants using a hydrogel-simulated model of the cell mechanical microenvironment of KC. The stiffness of the KC matrix microenvironment in vitro was 16.70 kPa and the stiffness of the normal matrix microenvironment was 34.88 kPa. Human keratocytes (HKs) were cultured for 24 h before observation or drug treatment with H_2_O_2_ in the presence or absence of SF. The levels of oxidative stress, nuclear factor E2-related factor 2 (Nrf-2) and antioxidant response element (ARE) were detected. The high-stress state of HKs in the mechanical microenvironment of KC cells compensates for the activation of the Nrf-2/ARE signaling pathway. H_2_O_2_ leads to increased oxidative stress and decreased levels of antioxidant proteins in KC. In summary, SF can reduce endogenous and exogenous oxidative stress and increase the antioxidant capacity of cells.

## Introduction

Keratoconus (KC) is a progressive degenerative disease that usually occurs bilaterally and is characterized by corneal thinning and apical protrusion of the cornea^1^. KC is a major clinical problem worldwide, with a reported prevalence of approximately 1 in 450 individuals^2^. The pathogenesis of KC remains unknown but may involve genetic and environmental factors^3,4^. However, accumulating evidence has revealed the role of oxidative stress in KC^5–8^. Oxidative stress is an indicator of the accumulation of reactive oxygen species (ROS), and KC keratocytes exhibit increased ROS production compared with that of normal keratocytes^6,7^. In vitro studies with pathway prediction software indicate that many altered pathways in KC keratocytes are related to oxidative stress^9^. Therefore, oxidative stress in KC keratocytes may play a major role in the development and progression of KC. However, the role of oxidative stress in KC remains unclear.

Nuclear factor E2-related factor 2 (Nrf-2) plays a key role in antioxidant response element (ARE)-regulated gene expression^10^ by binding to AREs and transactivating downstream target genes. The ARE-regulated expression of phase II detoxifying antioxidants and enzymes, including heme oxygenase-1 (HO-1), helps counteract increased oxidative stress and maintain redox status in many tissues^11,12^. As shown in our previous study, type II collagenase is useful for establishing an animal model of KC expansion both in vitro and in vivo^13,14^. The present study provides evidence that activation of the Nrf-2/ARE pathway partially promotes the protective effect of the antioxidant sulforaphane (SF) in the KC cornea^15^. However, to our knowledge, the effect of Nrf-2/ARE at the cellular level has not been studied in the cornea of patients with KC.

The microenvironment of the extracellular matrix is complex and diverse and plays an important role in regulating cell behavior. The cornea, a viscoelastic material, possesses unique biomechanical properties; thus, the mechanical microenvironment plays a crucial role in determining the behavior of keratocytes^16^. Small-magnitude stretching may promote corneal matrix synthesis, whereas large-magnitude stretching promotes corneal matrix degradation^17^. Based on these findings, changes in the corneal mechanical microenvironment may affect keratocytes. The mechanical microenvironment plays an important role in regulating the biological behavior of cells, such as cardiomyocytes^18^ and skeletal muscle cells^19^, leading to myocardial fibrosis, abnormal myotube formation and other diseases. Thus, studies aiming to clarify the pathological mechanism and prevent KC by constructing the mechanical microenvironment of keratocytes are very important.

Herein, we constructed a mechanical microenvironment based on hydrogel materials to explore the effects of microenvironmental changes on the function of keratocytes^20,21^. In vitro models of normal corneal and KC microenvironments were first generated. Then, human keratocytes (HKs) were cultured to detect changes in oxidative stress-related factors and the Nrf-2/ARE pathway and to further explore the role of keratocytes in the pathogenesis of KC. Keratocytes in the KC mechanical microenvironment were treated with SF to investigate changes in their biological behavior. An in-depth study of the regulatory effects of SF on the Nrf-2/ARE pathway in keratocytes in the KC microenvironment was conducted, and SF was shown to exert a protective effect on oxidative stress, providing a strategy for the early prevention and treatment of KC and other corneal dilatation diseases.

## Results

### Modeling KC mechanical characterization in vitro with polyacrylamide (PA) hydrogels

**Figure 1:**
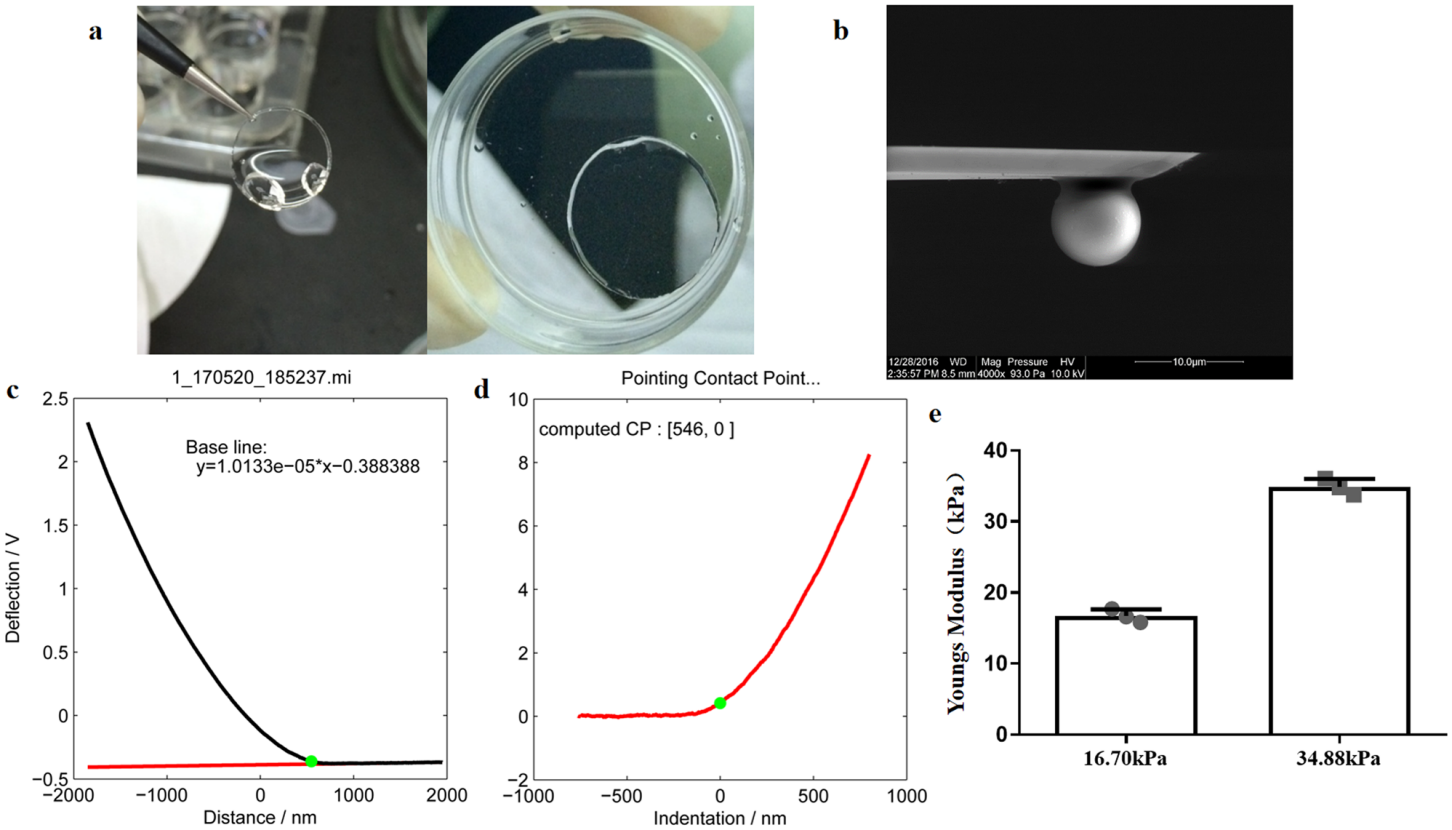
Stiffness measurement of the PA hydrogel. (**a**) A picture of the production of the PA hydrogel. (**b**) An AFM cantilever attached to a silica sphere. (**c**) Raw data of the representative force–distance curve (approach part). (**d**) A representative force–indentation curve converted from (**c**) by subtracting the measured deflection from the known response of the cantilever. (**e**) The stiffness of the PA hydrogels. The error bars indicate the SDs; n = 3 per group.

To simulate KC in vitro, we prepared PA hydrogels. As shown in Fig. 1, the stiffness of the two groups of PA hydrogels was measured using atomic force microscopy (AFM) after the hydrogels were successfully prepared. After the 2.5%/0.75% and 2.5%/1.5% acrylamide (%)/MBA (%) solutions were polymerized, the Young’s moduli were determined to be 16.70 ± 0.95 kPa and 34.88 ± 1.13 kPa, respectively. The final prepared PA hydrogel exhibited the expected stiffness.

**Figure 2:**
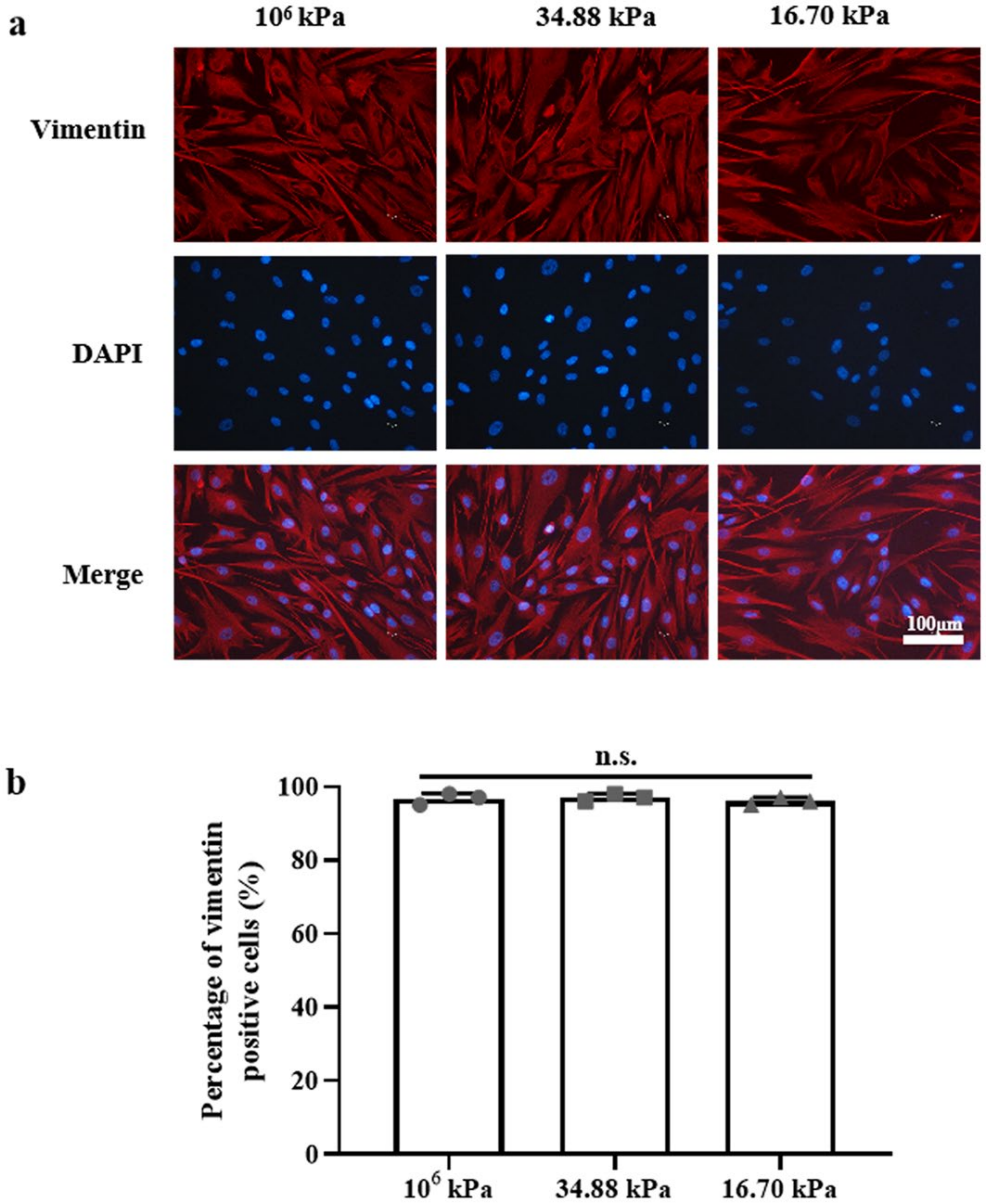
Human keratocytes (HKs) identification. (**a**) Detection of vimentin in HKs (immunofluorescence, × 200). Bar = 100 μm. DAPI, 4'-6-diamidino-2-phenylindole. (**b**) Percentage of vimentin-positive cells (%) under different culture conditions. Tukey's post hoc test was used to identify significant differences between samples. The data are presented as the means ± SDs; n = 3 per group. n.s., not significant.

HKs were cultured on three different substrates (10^6^ kPa, 34.88 kPa and 16.70 kPa) and showed a single layer of adherent growth. The cell bodies were star-shaped or spindle-shaped, with an oval-shaped nucleus in the center of each cell. Vimentin^+^ staining (Fig. 2) confirmed that the cells cultured in vitro were HKs. The morphology of the HKs in the 16.70 kPa group, which represents the KC mechanical microenvironment, did not change.

Changes in the mechanical microenvironment induce oxidative stress in keratocytes in PA hydrogels.

**Figure 3:**
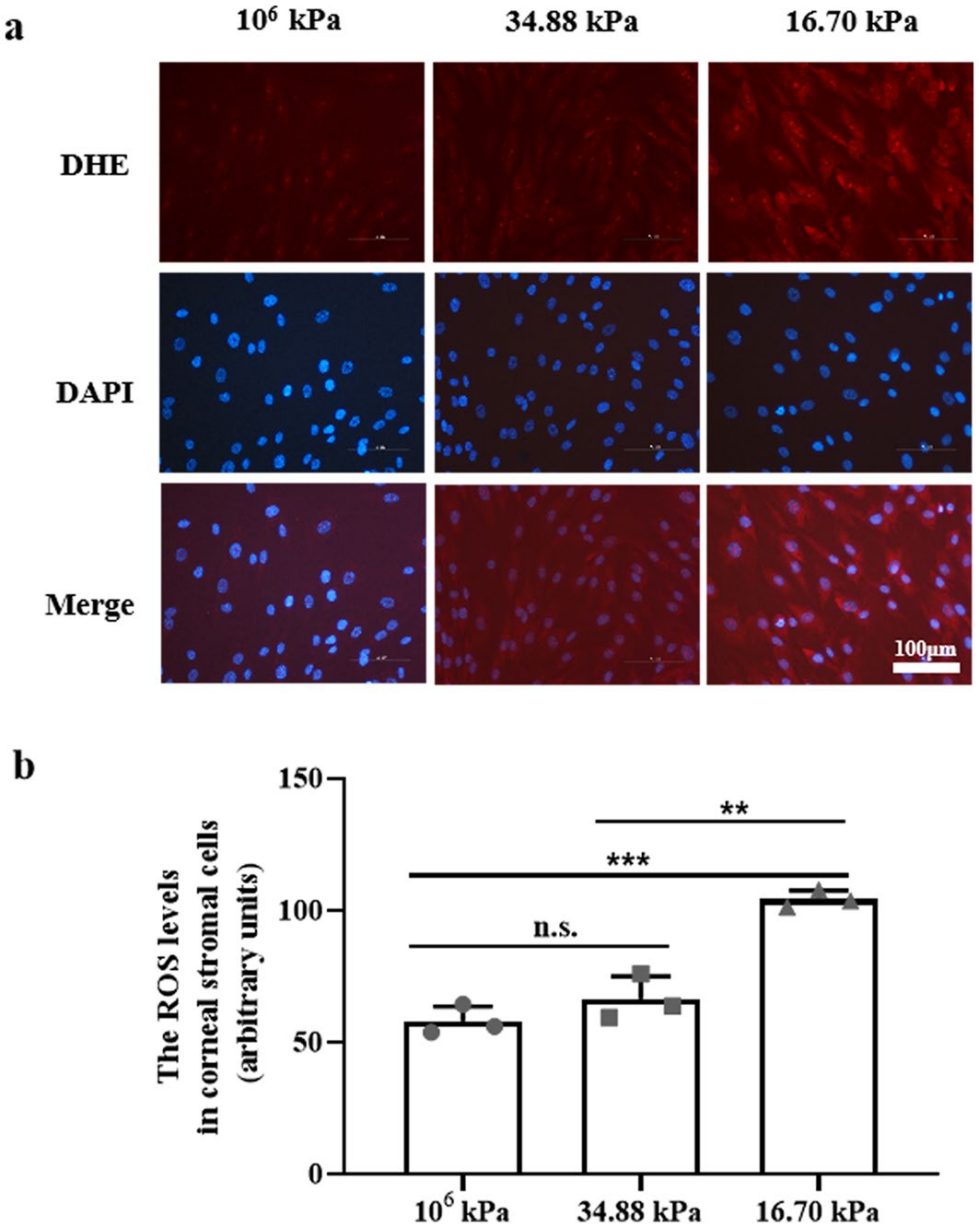
Generation of ROS in HKs. (**a**) The production of ROS in HKs was detected by DHE staining. Bar = 100 μm. (**b**) ROS levels in HKs. Tukey's post hoc test was used to identify significant differences between samples. The data are presented as the means ± SDs; n = 3 per group. **P < 0.01, ***P < 0.001, n.s., not significant.

**Figure 4:**
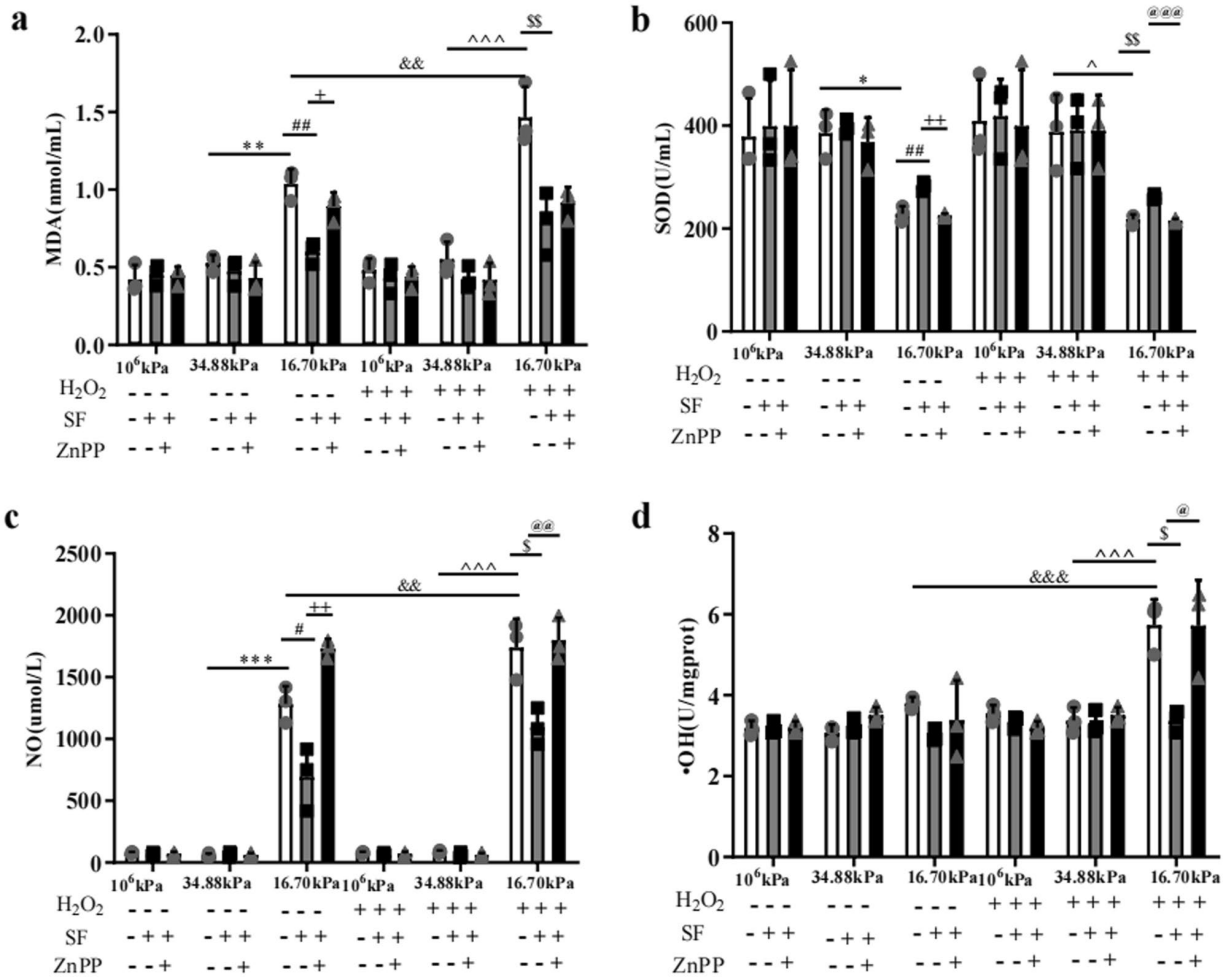
Detection of oxidative stress-related indicators under different culture conditions. (**a**) MDA content. (**b**) SOD. (**c**) NO. (**d**) ·OH. Tukey's post hoc test was used to identify significant differences between samples. The data are presented as the means ± SDs; n = 3 per group. *P < 0.05, **P < 0.01, ***P < 0.001 compared to the 34.88 kPa group. ^#^*P* < 0.05, ^##^*P* < 0.01 compared to the 16.70 kPa group. ^+^*P* < 0.05, ^++^*P* < 0.01 compared to the 16.70 kPa-SF group. ^&&^*P* < 0.01, ^&&&^*P* < 0.001 compared to the 16.70 kPa group. ^*P* < 0.05, ^^^*P* < 0.001 compared to the 34.88 kPa-H_2_O_2_ group. ^$^*P* < 0.05, ^$$^*P* < 0.01 compared to the 16.70 kPa-H_2_O_2_ group. ^@^*P* < 0.05, ^@@^*P* < 0.01, and ^@@@^*P* < 0.001 compared to the 16.70 kPa-H_2_O_2_-SF group.

To demonstrate whether changes in the mechanical microenvironment could induce oxidative stress in HKs in PA hydrogels, we measured the levels of malondialdehyde (MDA), superoxide dismutase (SOD), nitric oxide (NO), and hydroxyl radical (·OH). Based on immunofluorescence staining, the levels of detectable ROS were reduced only in the stromal cells in the 10^6^ kPa and 34.88 kPa groups (Fig. 3a), and the ROS level in the HKs in the KC microenvironment group was significantly greater than that in the 10^6^ kPa and 34.88 kPa groups (*P* < 0.001, *P* < 0.01; Fig. 3b). We measured the levels of MDA, SOD, NO, and ·OH to evaluate oxidative stress in the HKs of the three groups (Fig. 4). Significantly greater levels of MDA and NO and significantly lower SOD levels were detected in HKs within the 16.70 kPa group than the 34.88 kPa group.

**Figure 5:**
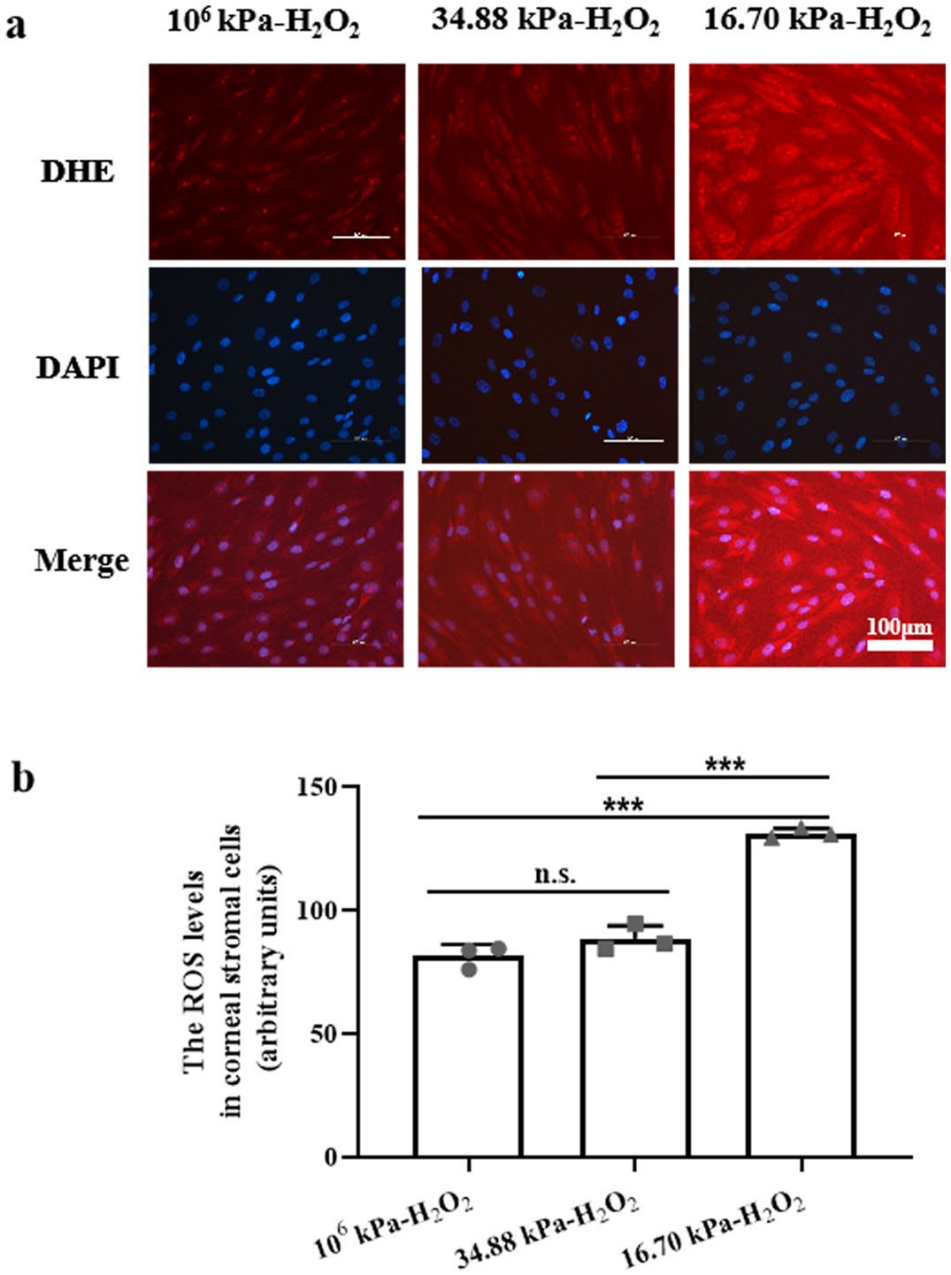
Generation of ROS in HKs after H_2_O_2_ treatment. (**a**) DHE staining was performed to detect the production of ROS. (b) ROS levels in corneal stromal cells. Tukey's post hoc test was used to identify significant differences between samples. The data are presented as the means ± SDs; n = 3 per group. ***P < 0.001, n.s., not significant.

The cells cultured on the three substrates were subjected to H_2_O_2_ treatment. As shown in Fig. 5, immunofluorescence staining revealed that the level of ROS in HKs in the 16.70 kPa-H_2_O_2_ group was significantly greater than that in the 10^6^ kPa and 34.88 kPa groups (both *P* < 0.001). HKs produced significantly greater levels of MDA, NO, and ·OH, and a significantly lower level of SOD was produced by HKs in the 16.70 kPa-H_2_O_2_ group than in the 34.88 kPa-H_2_O_2_ group (Fig. 4). Significantly greater levels of MDA, NO and ·OH were detected in the 16.70 kPa-H_2_O_2_ group than in the 16.70 kPa group (Fig. 4).

SF inhibits the oxidative stress response of HKs in the KC mechanical microenvironment.

To determine whether SF inhibited the oxidative stress response of HKs in the mechanical microenvironment of KC, we treated HKs in the 16.70 kPa/-H_2_O_2_ group with SF or SF + Znpp and compared the levels of MDA, SOD, NO, and ·OH levels. The MDA and NO levels were significantly lower and the SOD level was significantly greater in the 16.70 kPa-SF group than in the 16.70 kPa group (Fig. 4). Significantly greater MDA and NO levels and significantly lower SOD activity were detected in the 16.70 kPa-SF + Znpp group than in the 16.70 kPa-SF group (Fig. 4).

Cells in the 16.70 kPa-H_2_O_2_ group were treated with SF or SF + Znpp, and the levels of MDA, SOD, NO and ·OH were compared. The MDA, NO and ·OH levels were significantly lower and the SOD level was significantly greater in the 16.70 kPa-H_2_O_2_-SF group than in the 16.70 kPa-H_2_O_2_ group (Fig. 4). Znpp IX significantly increased NO and ·OH levels and decreased SOD levels (Fig. 4).

SF decreases the levels of Nox-2 and Nox-4 and increases the levels of Nrf-2 and HO-1 in HKs in the KC mechanical microenvironment.

**Figure 6:**
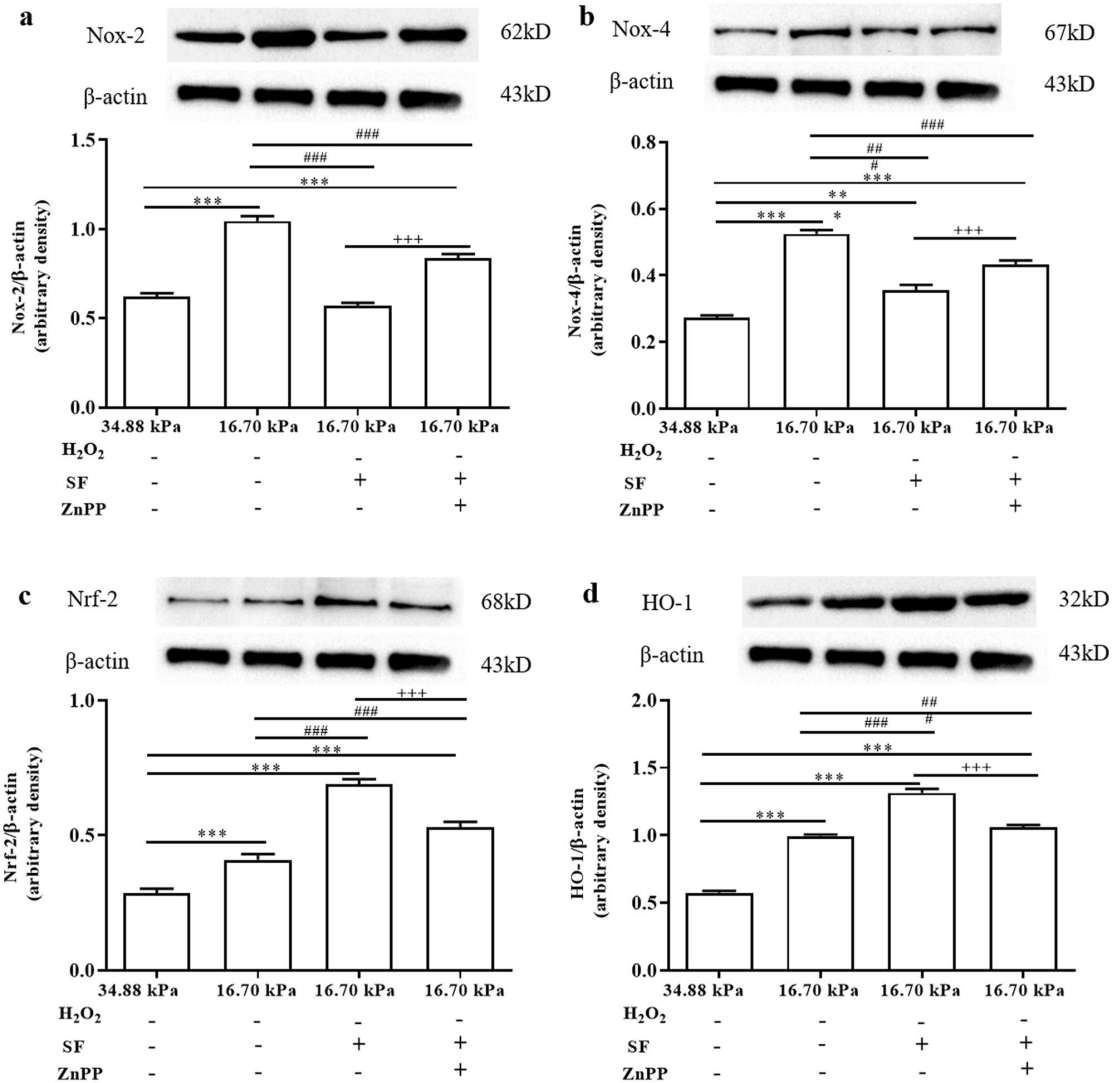
Expression levels of Nox-2, Nox-4, Nrf-2 and HO-1 in HKs under different experimental conditions. (**a**-**d**) The upper panels are protein grayscale images of Nox-2, Nox-4, Nrf-2 and HO-1 in Western blot analysis. The lower panels are the corresponding relative expression levels. The original blots are presented in S Figs. 5-9. Tukey's post hoc test was used to identify significant differences between samples. The data are presented as the means ± SDs; n = 3 per group. ***P < 0.001 compared to the 34.88 kPa group. ^###^*P* < 0.001 compared to the 16.70 kPa group; ^+++^*P* < 0.001 compared to the 16.70 kPa-SF group.

**Figure 7:**
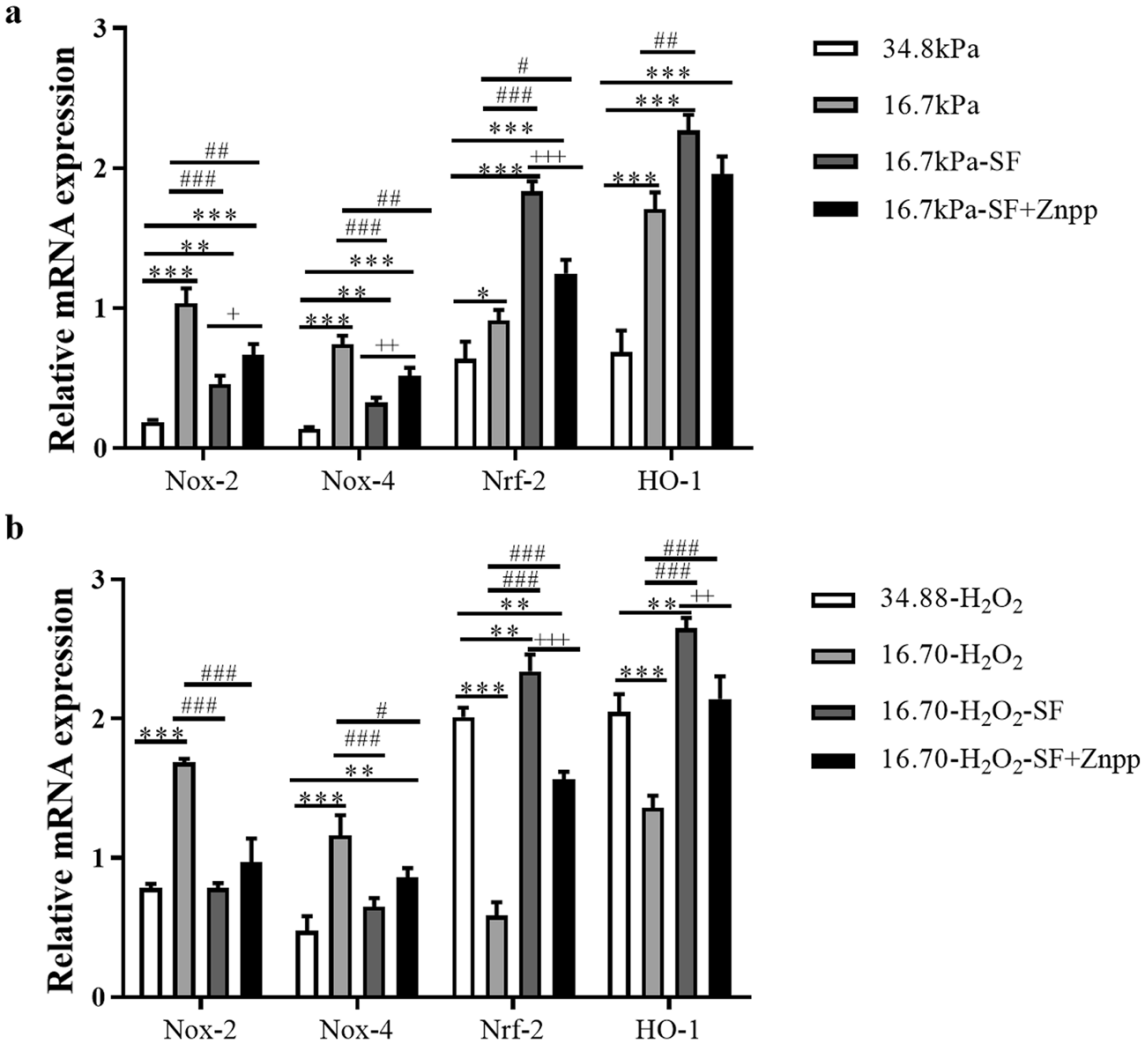
Determination of Nox-2, Nox-4, Nrf-2 and HO-1 mRNA levels under different experimental conditions. (**a**) SF downregulated Nox-2 and Nox-4 and upregulated Nrf-2 and HO-1 in HKs in the KC mechanical microenvironment. *P < 0.05, **P < 0.01, ***P < 0.001 compared to the 34.88 kPa group. ^##^*P* < 0.01, ^###^*P* < 0.001 compared to the 16.70 kPa group. ^+^*P* < 0.05, ^++^*P* < 0.01, ^+++^*P* < 0.001 compared to the 16.70 kPa-SF group. (**b**) SF downregulated Nox-2 and Nox-4 and upregulated Nrf-2 and HO-1 in HKs in the KC mechanical microenvironment after H_2_O_2_ treatment. **P < 0.01, ***P < 0.001 compared with the 34.88 kPa-H_2_O_2_ group. ^#^*P* < 0.05, ^###^*P* < 0.001 compared to the 16.70 kPa-H_2_O_2_ group. ^++^*P* < 0.01, ^+++^*P* < 0.001 compared to the 16.70 kPa-H_2_O_2_-SF group. The housekeeping gene used for normalization was β-actin. The fold change in gene expression relative to that of β-actin was calculated as 2^−ΔΔct^ (mean ± SD, n = 3). Tukey's post hoc test was used to identify significant differences between samples. The 34.88 kPa group, normal corneal mechanics microenvironment group. The 16.70 kPa group, KC mechanical microenvironment group.

To determine whether SF downregulates NADPH oxidase (Nox)-2 and Nox-4 levels and upregulates Nrf-2 and HO-1 levels in the KC mechanical microenvironment, we analyzed changes in the levels of proteins associated with oxidative stress in all groups by Western blot (WB) and RT‒qPCR analyses. The WB results showed that the levels of Nox-2 and Nox-4 proteins were significantly greater in the HKs in the 16.70 kPa group than the 34.88 kPa group (Fig. 6a, b). SF significantly reduced the levels of the Nox-2 and Nox-4 proteins, but these levels were significantly increased by Znpp IX. As shown in Fig. 6c and d, significantly greater levels of the Nrf-2 and HO-1 proteins were detected in HKs in the 16.70 kPa group than the 34.88 kPa group. SF significantly increased the levels of the Nrf-2 and HO-1 proteins, but Znpp IX significantly reduced their levels. RT‒qPCR quantitative analysis revealed a trend similar to that of the WB results (Fig. 7a).

SF upregulates Fn and COLI in HKs in the KC mechanical microenvironment.

To determine whether SF increases collagen deposition in the KC mechanical microenvironment, we conducted an experiment with Fn and COLI immunofluorescence. SF treatment increased the expression levels of Fn (S Fig. 1) and COLI (S Fig. 2) in keratocytes in the KC microenvironment, suggesting that SF is a therapeutic for KC.

SF downregulates Nox-2 and Nox-4 and upregulates Nrf-2 and HO-1 in HKs in the KC mechanical microenvironment after H_2_O_2_ treatment.

**Figure 8:**
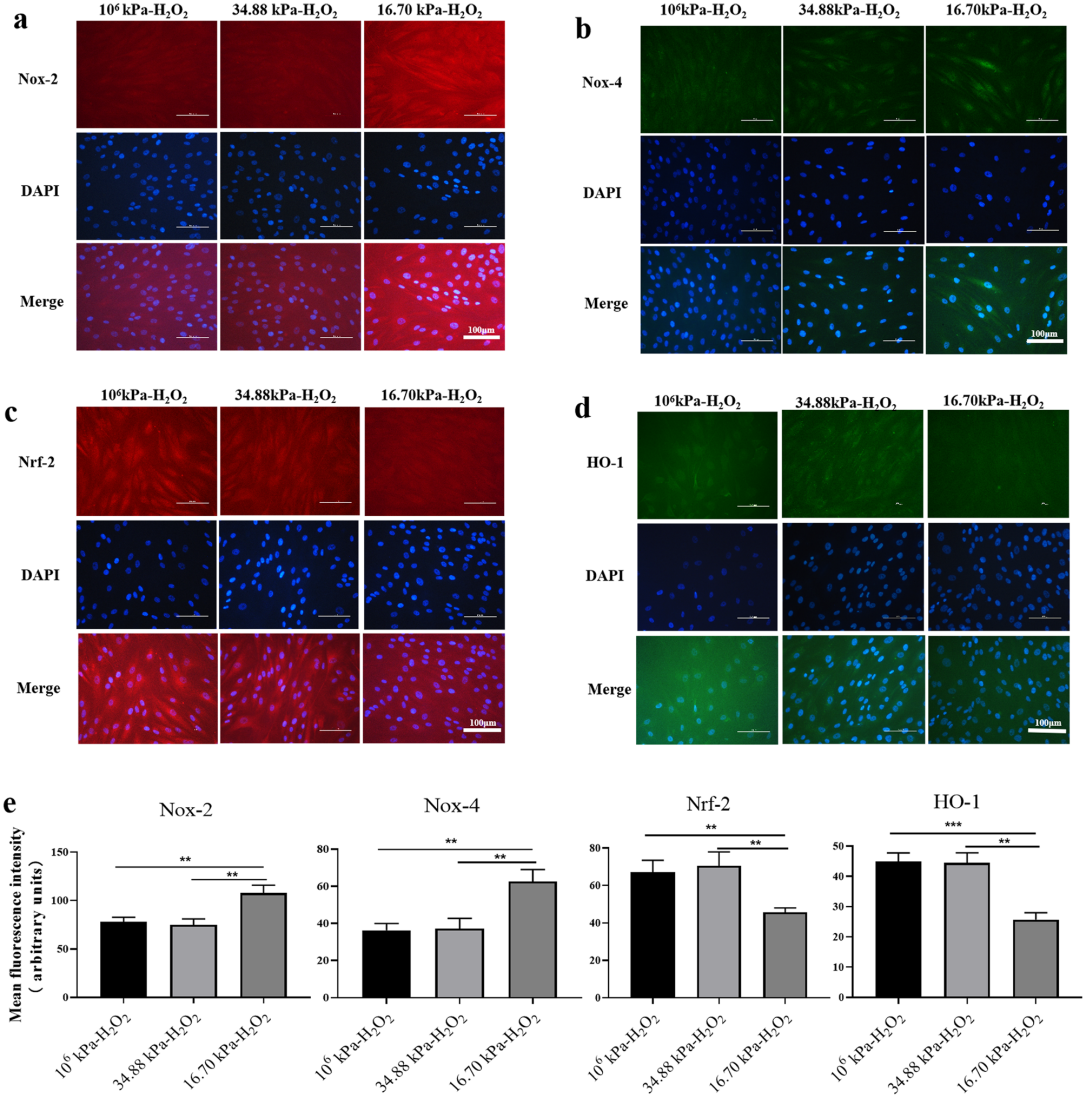
Evaluation of Nox-2, Nox-4, Nrf-2 and HO-1 expression in HKs by immunofluorescence after H_2_O_2_ treatment. (**a**-**d**) Representative micrographs of HKs obtained from each group stained with an anti-Nox-2 antibody (red, **a**), an anti-Nox-4 antibody (green, **b**), an anti-Nrf-2 antibody (red, **c**), and an anti-HO-1 antibody (green, **d**) are shown in the upper panels. The middle panels show the corresponding images of DAPI-stained sections (blue). The lower panels show merged images. Scale bar = 100 μm. DAPI, 4'-6-diamidino-2-phenylindole. (**e**) The mean fluorescence intensity of Nox-2, Nox-4, Nrf-2 and HO-1 in the experimental groups. The data are presented as the means ± SDs; n = 3 per group. **P < 0.01, ***P < 0.001. The 10^6^ kPa group, included conventional cell culture stiffness groups (the matrix stiffness of the rigid plastic dishes). The 34.88 kPa group, normal corneal mechanics microenvironment group. The 16.70 kPa group, KC mechanical microenvironment group.

**Figure 9:**
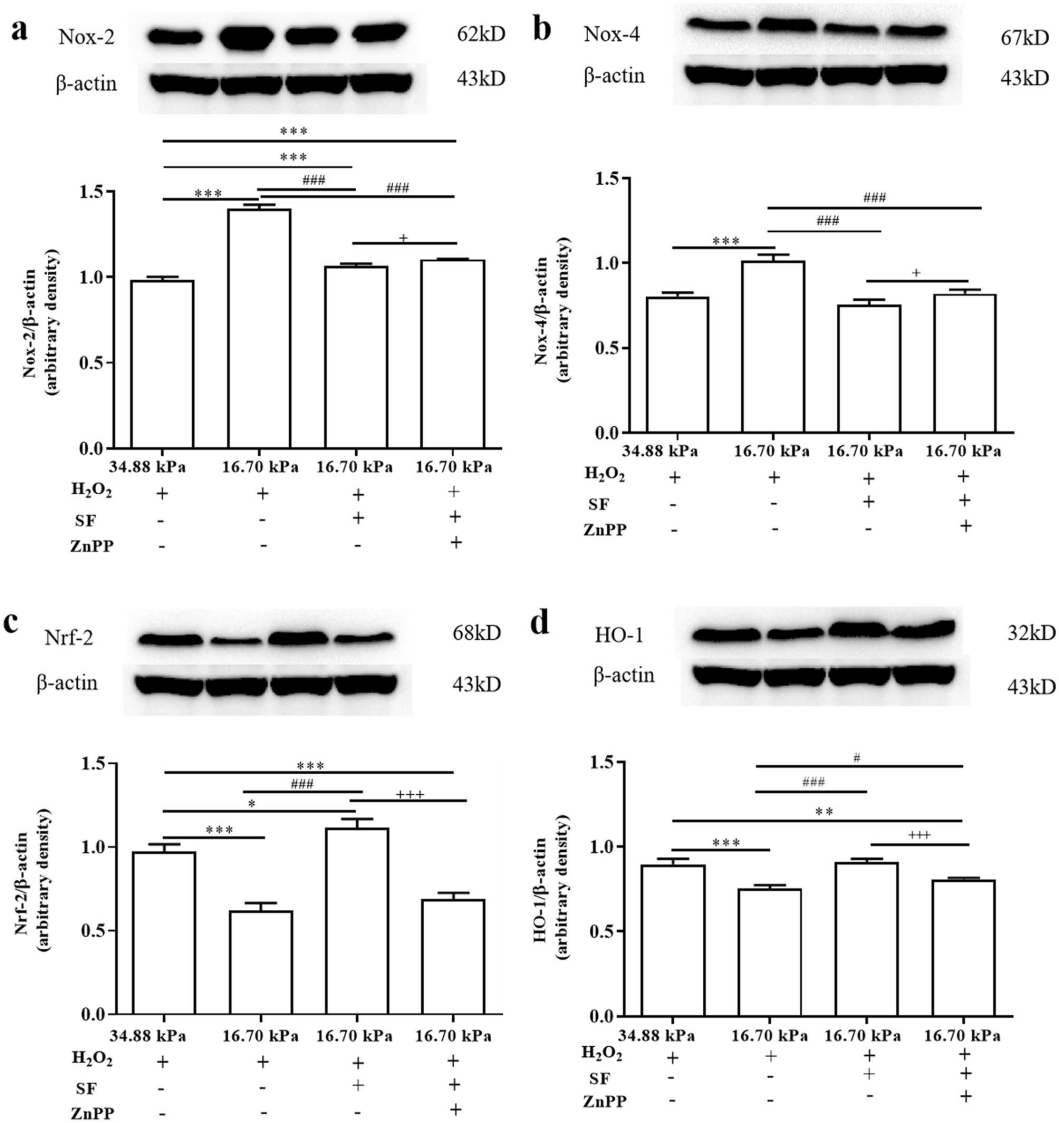
Expression levels of Nox-2, Nox-4, Nrf-2 and HO-1 in HKs under different experimental conditions after H_2_O_2_ treatment. (**a**-**d**) Representative immunoblotting showing the protein levels of Nox-2, Nox-4, Nrf-2 and HO-1 in HKs (upper panel) and densitometric analysis of the expression of Nox-2, Nox-4, Nrf-2 and HO-1 relative to the loading control (lower panel). The original blots are presented in S Figs. 10-14. Tukey's post hoc test was used to identify significant differences between samples. The data are presented as the means ± SDs, n = 3 per group. *P < 0.05, **P < 0.01, ***P < 0.001, vs. the 34.88 kPa-H_2_O_2_ group. ^#^*P* < 0.05, and ^###^*P* < 0.001, vs. the 16.70 kPa-H_2_O_2_ group. ^+^*P* < 0.05, ^+++^*P* < 0.001, vs. the 16.70 kPa-H_2_O_2_-SF group.

Next, we examined the expression of Nox-2, Nox-4, Nrf-2 and HO-1 in HKs in the 10^6^ kPa, 34.88 kPa and 16.70 kPa groups treated with H_2_O_2_ by immunofluorescence staining to evaluate the levels of proteins associated with oxidative stress in HKs in the KC mechanical microenvironment after H_2_O_2_ treatment (Fig. 8). Nox-2 and Nox-4 immunoreactivity was lower in HKs within the 16.70 kPa-H_2_O_2_ group than in HKs within the other two groups (Fig. 8a, b, e). The immunoreactivity of Nrf-2 and HO-1 in the 16.70 kPa-H_2_O_2_ group was greater than that in the other two groups (Fig. 8c, d, e). The levels of Nox-2, Nox-4, Nrf-2 and HO-1 in the 34.88 kPa-H_2_O_2_, 16.70 kPa-H_2_O_2_, 16.70 kPa-H_2_O_2_-SF, and 16.70 kPa-H_2_O_2_-SF + Znpp groups were analyzed. The WB results revealed that the levels of the Nox-2 and Nox-4 proteins were significantly greater in the 16.70 kPa-H_2_O_2_ group than in the 34.88 kPa-H_2_O_2_ group (Fig. 9a, b). SF significantly reduced the levels of the Nox-2 and Nox-4 proteins, but Znpp IX significantly increased their levels. As shown in Fig. 9c and d, significantly lower levels of the Nrf-2 and HO-1 proteins were detected in the 16.70 kPa-H_2_O_2_ group than in the 34.88 kPa-H_2_O_2_ group (all *P* < 0.001). SF significantly increased the levels of Nrf-2 and HO-1, but Znpp IX significantly reduced their levels. RT‒qPCR analysis revealed a trend similar to that of WB analysis (Fig. 7b).

## Discussion

In this study, PA hydrogels with stiffnesses mimicking the normal corneal (34.88 kPa) and KC (16.70 kPa) mechanical microenvironments were prepared^22–26^. The elastic modulus in KC is approximately half that of the normal cornea^24,25,27^. Additionally, we utilized coverslips with stiffness akin to conventional cell culture substrates (10^6^ kPa) as experimental controls^28^.

Oxidative stress has long been implicated in the pathogenesis of KC^29–34^. Our findings corroborate this, revealing elevated levels of ROS, MDA and NO in the KC microenvironment, alongside reduced SOD levels^29,35^. Abnormalities in matrix stiffness might significantly alter cell behavior, leading to disease^36–38^. As shown in the present study, H_2_O_2_ treatment exacerbated the oxidative stress response in HKs in the KC microenvironment, accompanied by an increase in ROS, MDA, NO and ·OH levels and a decrease in SOD levels. Notably, HKs cultured within the KC microenvironment exhibited heightened sensitivity to oxidative stress, indicating their potential role in the development and progression of KC.

In contrast to normal corneas, KC corneas do not activate the Nrf-2/ARE pathway^39–41^. Preliminary experiments revealed higher Nox-2 and Nox-4 expression but lower Nrf-2 and HO-1 levels in KC tissues compared to normal corneal tissue (S Fig. 3), suggesting limited activation of the antioxidant pathway in KC. A decrease in Nrf-2 may underlie the poor antioxidant functions in KC^14,42,43^. SF, an Nrf-2 activator, mitigates ROS accumulation in corneal stromal cells and enhances Nrf-2 nuclear translocation^39,44^. SF significantly reduced NO levels, increased SOD levels, and inhibited oxidative stress in HKs within the KC microenvironment. Additionally, SF inhibited the oxidative stress induced by H_2_O_2_, partially reversed by Znpp IX, an inhibitor of HO-1. Future gradient experiments with ZnPP IX concentration could elucidate HO-1 dependency. Overall, SF protects against oxidative stress in KC by targeting HKs, with HO-1 playing a crucial role.

Corneal stromal cells generate ROS through Nox-mediated NADPH oxidation, mainly via Nox-2 and Nox-4 activation^7,45^. Under oxidative stress, Nrf-2 translocates to the nucleus, prompting the expression of AREs such as HO-1^46^. In the present study, Nox-2 and Nox-4 levels were significantly increased in HKs within the KC microenvironment, indicating their involvement in ROS production. Increased Nrf-2 and HO-1 levels in KC imply an endogenous mechanism against oxidative stress. However, interpreting these findings in KC is challenging without pathological cells, and mechanical tension inadequately models KC, limiting our study. Additionally, ROS levels were notably higher in KC-derived cells compared to normal corneal stromal cells (S Fig. 4), indicating their heightened susceptibility to oxidative damage in response to external stimuli. This underscores the physiological role of ROS in activating Nrf-2, thereby mediating SF's protective effect against oxidative stress in the KC microenvironment, through the Nrf-2/ARE pathway.

Studies indicate a decrease in total antioxidant capacity in KC, potentially associated with heightened oxidative stress and tissue damage^47,48^. H_2_O_2_ notably increased Nox-2, Nox-4, and ROS levels while decreasing Nrf-2 and HO-1 levels in the KC microenvironment. Interestingly, HKs in the KC microenvironment didn't adaptively regulate H_2_O_2_-induced oxidative stress or activate the Nrf-2/ARE pathway, contrasting in vivo findings. SF significantly downregulated Nox-2 and Nox-4 and upregulated Nrf-2 and HO-1 in the KC microenvironment after H_2_O_2_ treatment. This suggests that SF induces HO-1 expression, facilitates Nrf-2 nuclear translocation, activates the Nrf-2/ARE pathway, and exerts antioxidant effects on HKs in the KC microenvironment following H_2_O_2_ treatment.

Our study has several limitations. First, further investigation is needed to simulate the stiffness of the KC matrix microenvironment and explore its effects. on HKs. Second, correlating the stiffness of KC stromal cells with the microenvironment could aid in the early diagnosis of KC. In addition, studying cells in a 3D microenvironment could better simulate in vivo conditions and enhance our understanding of the biological behavior of HKs.

In conclusion, the high-stress state of HKs in the KC mechanical microenvironment compensates for Nrf-2/ARE pathway activation. H_2_O_2_-induced oxidative stress decreases antioxidant protein expression in KC. SF mitigates both endogenous and exogenous oxidative stress and enhances cellular antioxidant capacity, closely linked to Nrf-2/ARE activation. Our findings lay the groundwork for SF's potential as a therapeutic agent for KC.

## Materials and methods

### Fabrication of PA hydrogels

Glass coverslips and slides were cleaned to remove organics, dried in an oven and functionalized with 2% 3-(trimethoxysilyl) propyl methacrylate. Moreover, the surface of the glass slide was treated with dichloromethylsilane (DCMS). The PA hydrogels were fabricated according to protocols described elsewhere^26,49^. Briefly, a polymer solution containing acrylamide, MBA (N,N-methylene-bis-acrylamide, Sigma Aldrich, St. Louis, USA), 10% aluminum persulfate and a 1/1000 volume of tetramethylethylenediamine was prepared. This solution was sandwiched between a DCMS-treated slide and a functionalized coverslip. Following polymerization, the PA hydrogel was incubated with 1 mg/mL sulf-SANPAH, washed with 50 mM 4-(2-hydroxyethyl)-1-piperazineethanesulfonic acid buffer at pH 8.5 and then incubated with 0.12 mg/mL rat tail collagen type I overnight at room temperature. The dishes were placed on the sample stage of a custom bio-AFM system equipped with an inverted microscope (Nikon, TE2000U). All AFM data were analyzed using SPIP 6.3.3 software to determine the Young’s modulus, which represents the stiffness of the PA hydrogel.

### Cell culture and treatment

HKs were acquired from ScienCell Research Laboratories (catalog #6520) and cultured in fibroblast medium. All cells on the hydrogel substrate and on common culture dishes were cultured for 24 h before detection or drug treatment. HKs were treated with SF (50 μM, 4 h) (Sigma) in the presence or absence of 10 μM Znpp IX (a specific inhibitor of HO-1, 1 h) (Sigma). For oxidative stress analysis, HKs were treated with 200 μM H_2_O_2_ for 1 h with or without 50 μM SF for 4 h and 10 μM Znpp IX for 1 h. The treatment concentrations used in our study were evaluated and optimized in our primary experiments. Cells from the third to fifth passages were utilized in this study.

### Analysis of ROS generation

The generation of corneal ROS was assessed using dihydroethidium (DHE; Invitrogen Molecular Probes, Eugene, OR) as previously described^50^. Briefly, HKs were carefully washed with sterile PBS after the cell culture medium was removed. The cells were incubated with 5 μM DHE at 37 °C for 30 min and rinsed 2–3 times with sterile PBS. The cells were kept away from light during the procedure and were sealed with a sealing sheet. Green light was emitted under a fluorescence microscope, and the red emission of corneal stromal cells was observed and imaged.

### Immunocytochemistry

HKs were seeded onto coverslips, and PA hydrogels were added to the wells of a six-well plate (5 × 10^5^ cells/well). Next, the cells were fixed with 4% paraformaldehyde, permeabilized with 0.3% Triton X-100 for 20 min, and then blocked with 2% normal goat serum. The cells were then incubated overnight at 4 °C with primary antibodies against HO-1 (ADI-SPA-895-F, 1:100), Nox-2 (ab80508, 1:1000), Nox-4 (NB110-58,849, 1:500), Nrf-2 (bs-1074R, 1:500), vimentin (ab192890, 1:400), Fn (NBP1-51,723, 1:400) and COLI (NB600-408, 1:400). The cells were incubated with secondary antibody for 1 h at 25 °C, and their nuclei were stained with DAPI. Finally, the stained cells on the coverslips were observed under an Olympus optical microscope.

### Western blotting

Total protein was extracted from the cells by sonication in RIPA buffer (C1053, Applygen, Beijing, China). A bicinchoninic acid protein assay reagent kit (P1511, Applygen) was used to measure the protein concentration (n = 5 per group). Equal amounts of protein (20 µg/lane) were separated by 10 or 12% SDS‒PAGE. Next, the proteins were transferred to an Immun-Blot PVDF membrane that was subsequently blocked and probed with one of the following primary antibodies: HO-1, Nox-2, Nox-4, Nrf-2 or mouse monoclonal anti-β-actin (A1978, 1:2000). Protein bands were visualized using Amersham Biosciences ECL Western blot detection reagent (GE Healthcare Life Sciences, Uppsala, Sweden). For quantification, blots from at least three independent experiments were used and quantified using ImageJ software.

### Measurement of the MDA, SOD, NO, and ·OH levels

The levels of MDA, SOD, NO, and ·OH in the samples at the end of the treatments were quantified by utilizing a commercial kit according to the manufacturer’s instructions (Abcam, MA, USA).

### Real-time PCR

To evaluate mRNA expression, RT‒PCR was performed on all samples as previously described^51^. Briefly, total RNA extraction was carried out using an Ambion RNA Mini Extraction Kit. cDNA synthesis was performed using a PrimeScript RT reagent kit (RR047A, TaKaRa Bio Inc., Japan). The quality and concentration of the synthesized cDNA were measured with an ND-1000 Nanodrop spectrophotometer. RT‒PCR was performed with SYBR Premix Ex Taq II (RR820A, TaKaRa). The reaction contained 10 ng of cDNA in a 20-μl volume using a PikoReal 96 real-time PCR system (Thermo, Waltham, MA, USA). The conditions used were two minutes at 50 °C, 30 s at 94 °C, and 40 cycles of 5 s at 94 °C and 34 s at 60 °C. The expression of each gene was quantified in duplicate. The housekeeping gene used for normalization was β-actin. The relative mRNA expression was normalized against the expression of β-actin and calculated using the 2^−ΔΔct^ method. The primers used are shown in Table 1 (AuGCT DNA-SYN Biotechnology). GraphPad Prism 6 and MS Excel were used for data analysis. All samples and analyses were repeated at least three times.

**Table 1:** Gene markers and corresponding primers.

Gene	Forward sequence (5’-3’)	Reverse sequence (5’-3’)
HO-1	CTTCTTCACCTTCCCCAACA	GCTCTGGTCCTTGGTGTCAT
Nox-2	TGCACCTCAGGTATCAATTC	AAGGGCCAATATTCTCAGAC
Nox-4	GGAGCAATAAGCCAGTCACC	GAACCCCAAATGTTGCTTTG
Nrf-2	ACCATGGTTCCAAGTCCAGA	CTGTCAACTGGTTGGGGTCT
β-actin	TGACGTGGACATCCGCAAAG	TCTTCATTGTGCTGGGTGCC

### Statistical analysis

All results are expressed as the mean ± standard deviation (SD). The results of multiple groups were compared using one-way analysis of variance followed by Tukey's post hoc test using SPSS Statistics, version 17.0 (SPSS, Inc., Armonk, NY) unless otherwise noted. Differences were considered significant at *P* < 0.05. In addition, n = 3 unless otherwise noted. No statistical methods were used to predetermine the sample size.

### Supplementary Information

Supplementary Information.

## Data Availability

The data that support the findings of this study are available from the corresponding author [yanxiaoming7908@163.com] upon reasonable request.
