# Supplementary material for: Balanced activation of Nrf-2/ARE mediates the protective effect of sulforaphane on keratoconus in the cell mechanical microenvironment
Source: Sci Rep. 2024 Mar 23;14:6937. doi: 10.1038/s41598-024-57596-9 (PMC10960822; doi:10.1038/s41598-024-57596-9)
Supplement: Supplementary file 1 — Supplementary Information. [file 41598_2024_57596_MOESM1_ESM.pdf]

Supporting Information

Balanced Activation of Nrf-2/ARE Mediates the Protective Effect of Sulforaphane on Keratoconus in the Cell Mechanical Microenvironment

Ruixing Liu [1, 2], Ruojun Ma [2], Xiaoming Yan [1]*

[1] *Department of Ophthalmology, Peking University First Hospital, Beijing, People's Republic of China*
[2] People's Hospital of Zhengzhou University, Henan Provincial People's Hospital, Henan Eye Hospital, Zhengzhou 450003, China
****Correspondence to:*** *Xiaoming Yan, Department of Ophthalmology, Peking University First Hospital, Beijing 100034 P. R. China; Tel: +86-10-83573417; Fax: +86-10-83572748; e-mail: yanxiaoming7908@163.com.*

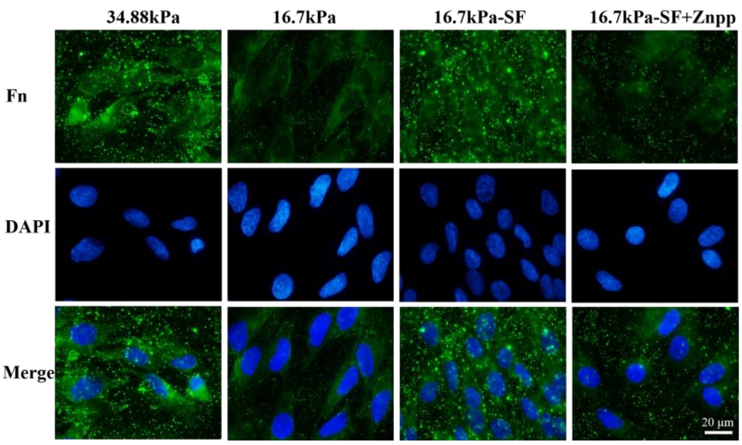

S Fig. 1. The expression of Fn in keratocytes under different experimental conditions.

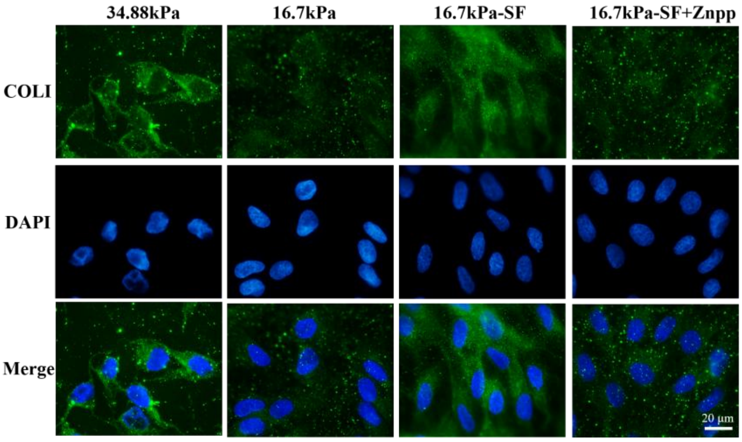

S Fig. 2. The expression of COLI in keratocytes under different experimental conditions.

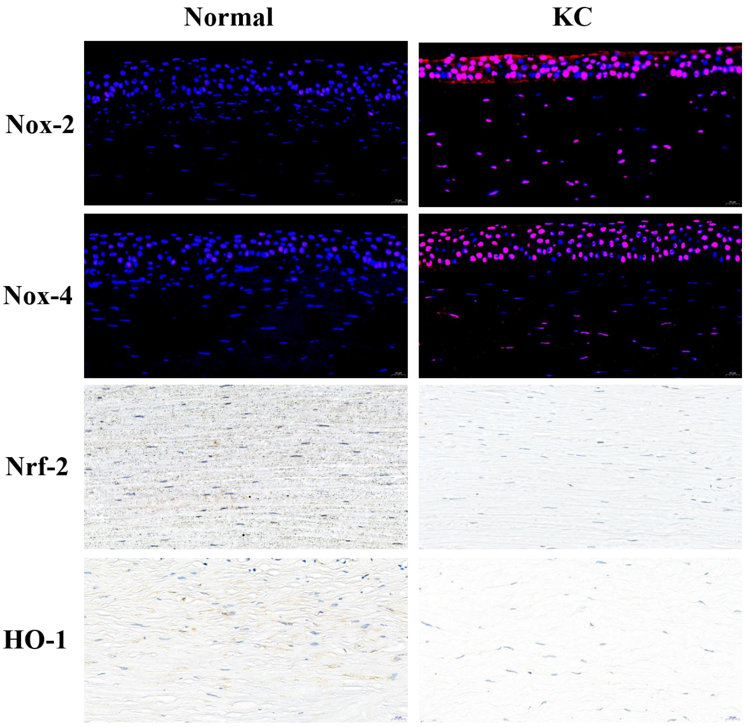

S Fig. 3. The expression of Nox-2, Nox-4, Nrf-2 and HO-1 in normal corneas (Normal) and KC tissues (KC).

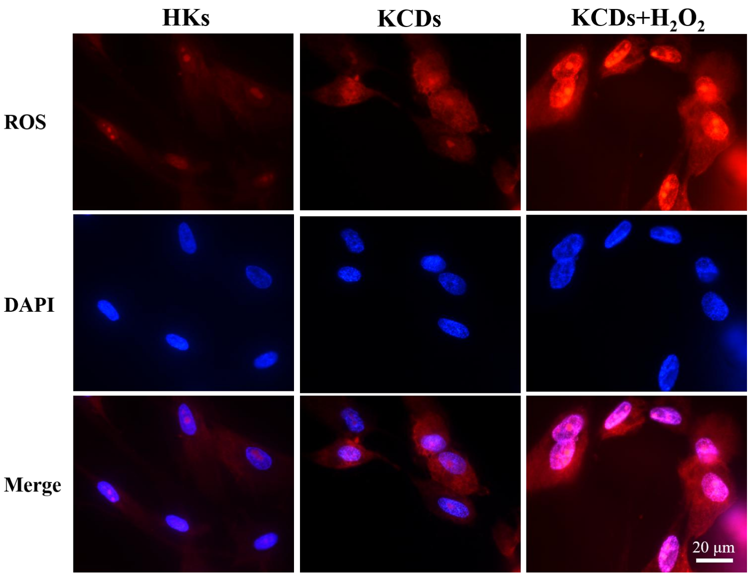

S Fig. 4. The levels of ROS in human keratocytes (HKs) and KC-derived cells (KCDs) treated with or without H_2O_2.

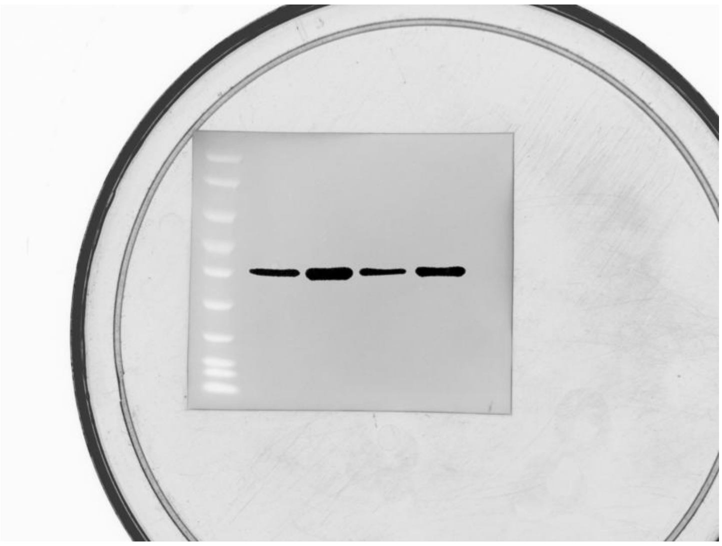

S Fig. 5. The Nox-2 for Figure 6A.

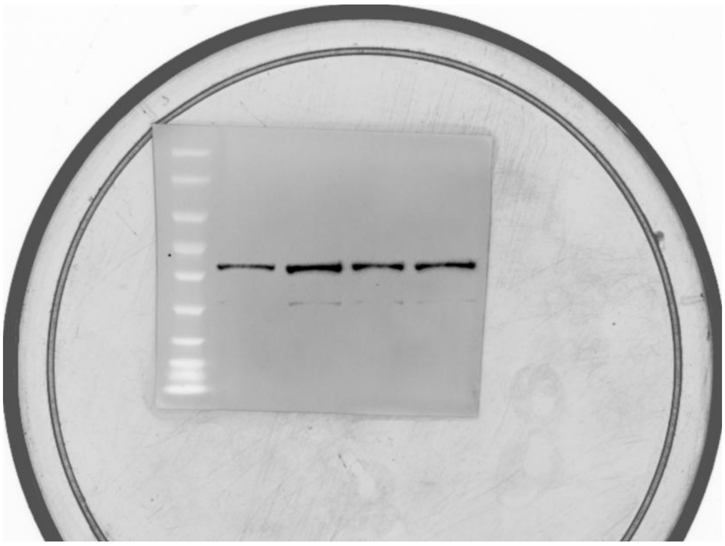

S Fig. 6. The Nox-4 for Figure 6B.

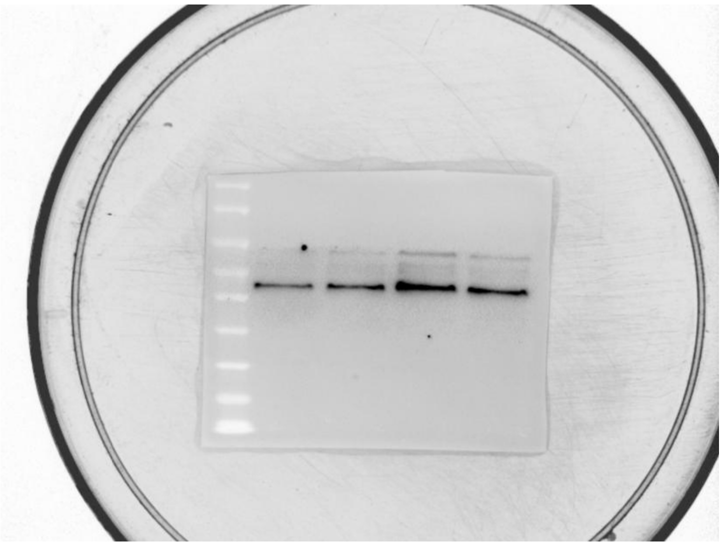

S Fig. 7. The Nrf-2 for Figure 6C.

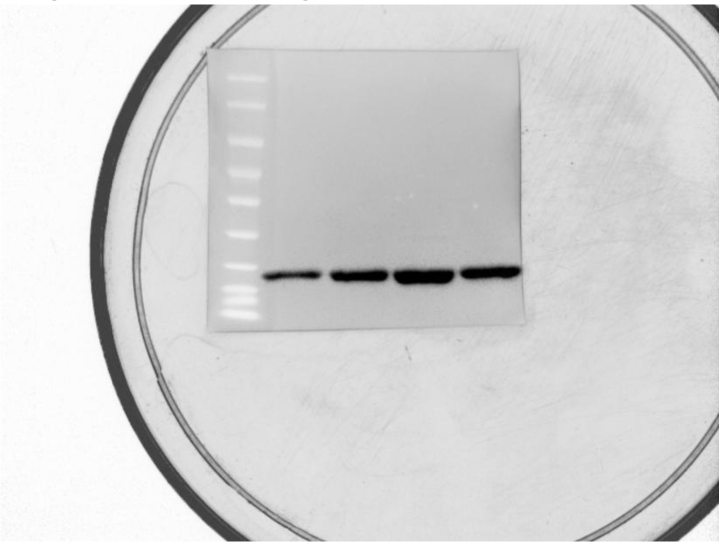

S Fig. 8. The HO-1 for Figure 6D.

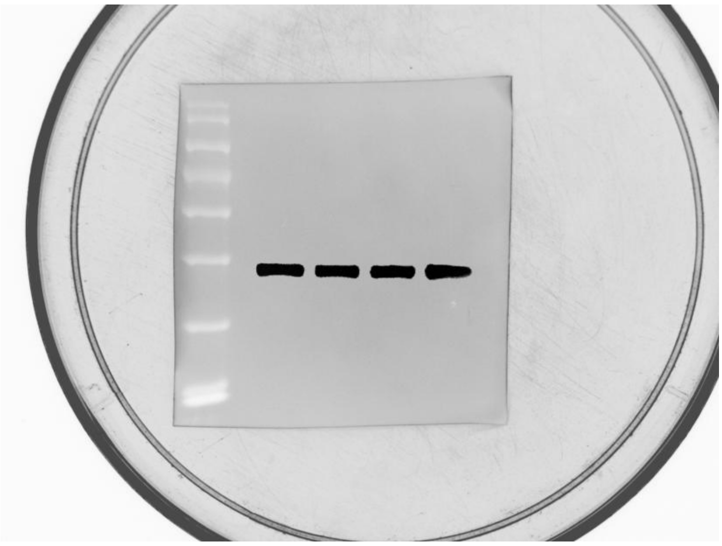

S Fig. 9. The β-actin for Figure 6.

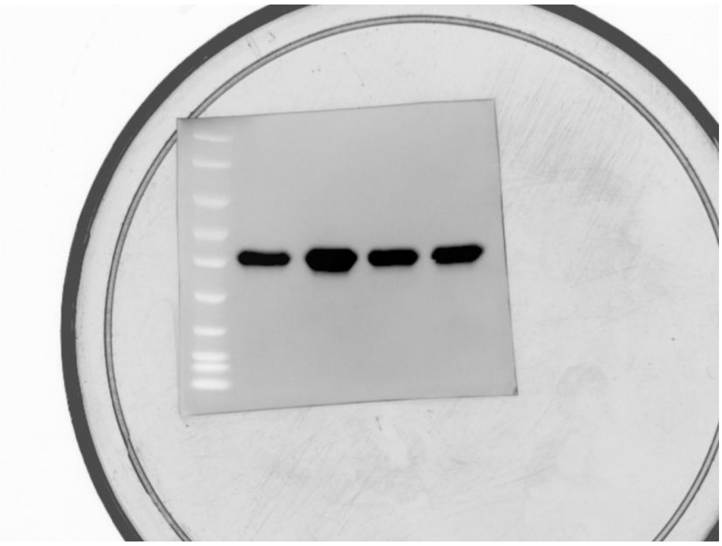

S Fig. 10. The Nox-2 for Figure 9A.

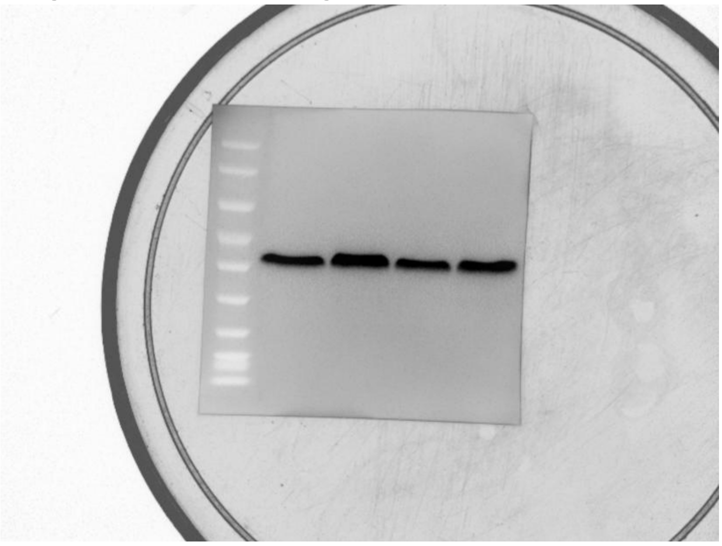

S Fig. 11. The Nox-4 for Figure 9B.

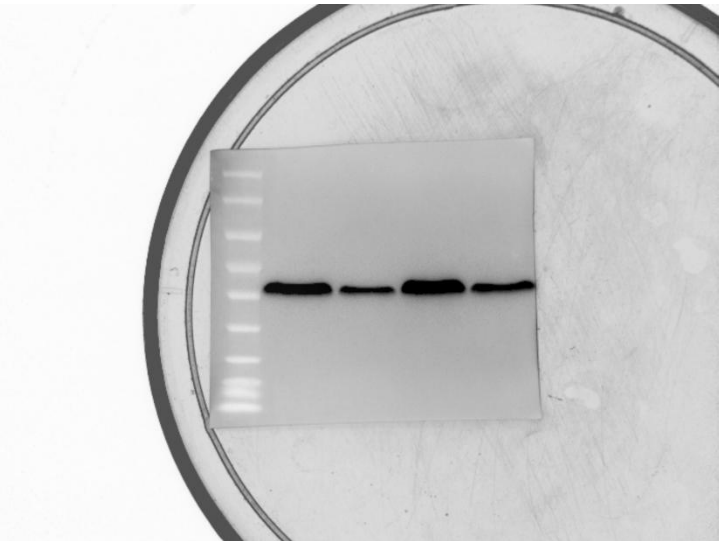

S Fig. 12. The Nrf-2 for Figure 9C.

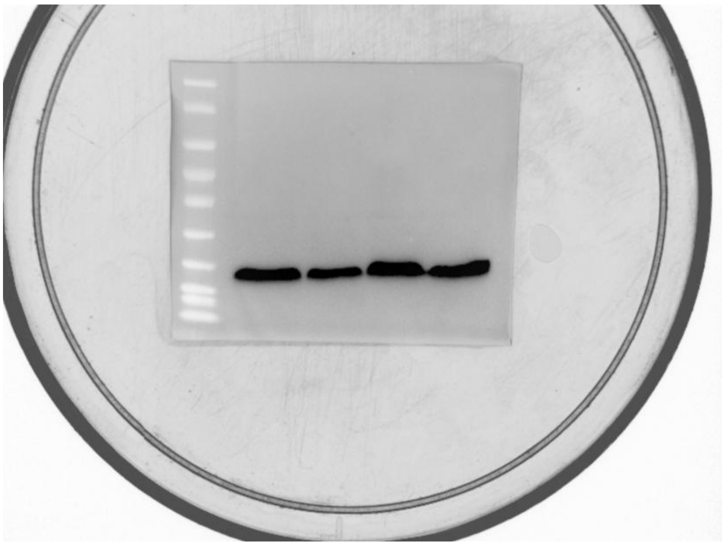

S Fig. 13. The HO-1 for Figure 9D.

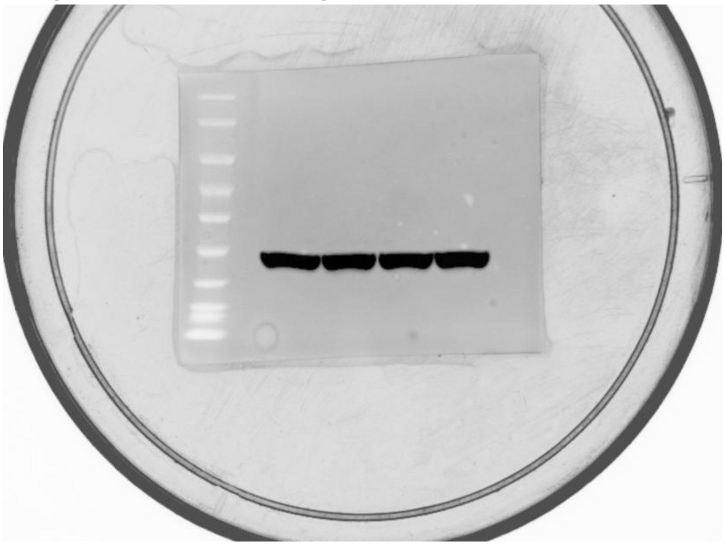

S Fig. 14. The β-actin for Figure 9.
